# Pharmacokinetics of rituximab in a pediatric patient with therapy-resistant nephrotic syndrome

**DOI:** 10.1007/s00467-015-3120-8

**Published:** 2015-06-09

**Authors:** Clare E. Counsilman, Cornelia M. Jol–van der Zijde, Jasper Stevens, Karlien Cransberg, Robbert G. M. Bredius, Ram N. Sukhai

**Affiliations:** Department of Pediatrics, Leiden University Medical Center (LUMC), Albinusdreef 2, 2333 ZA Leiden, The Netherlands; Department of Pediatric Nephrology, Erasmus MC Sophia, Rotterdam, The Netherlands; Center for Human Drug Research, Leiden, The Netherlands

## Abstract

**Background:**

Rituximab (RTX) has recently been introduced as a second-line therapy for nephrotic syndrome in children. Studies show that RTX given during the nephrotic state may be less effective than treatment during a non-nephrotic state, possibly due to loss of RTX in the urine.

**Case-Diagnosis/Treatment:**

We describe a 10-year-old boy with steroid-resistant nephrotic syndrome (SRNS) treated with RTX during a phase of active non-selective proteinuria. The serum half-life of RTX in this patient was less than 1 day compared to 20 days in patients without protein losses. Urinary clearance was at least 25 %, compared to approximately 0 % in control patients. However, RTX loss in the urine, as well as in pleural effusion and ascites, only partly explains the rapid drop in the serum RTX concentration of this patient.

**Conclusions:**

Serum half-life of RTX can be extremely short, partly due to excessive urinary losses in therapy-resistant nephrotic syndrome with non-selective proteinuria, as seen in our patient. These findings may help to explain the poor results of RTX treatment in patients with active proteinuria.

**Electronic supplementary material:**

The online version of this article (doi:10.1007/s00467-015-3120-8) contains supplementary material, which is available to authorized users.

## Background

Over the last few years rituximab (RTX), a humanized monoclonal antibody against CD20+ B cells, has been introduced as a second-line therapy for nephrotic syndrome in children non-responsive to standard therapies. B cells produce (auto)-antibodies, generate pro-inflammatory cytokines and function as modulators of inflammation, and are efficient antigen-presenting cells [1]. Although nephrotic syndrome is thought to be primarily a T cell-mediated disease, anti-B cell therapy probably has an immunomodulatory effect on the disease course [2, 3].

Several studies have shown that RTX given during the nephrotic state may be less effective than treatment during a non-nephrotic state (i.e., in remission) [2, 4, 5], possibly due to the loss of RTX in the urine [2, 5–7]. However, to date, there has been no pharmacokinetics-based evidence for this hypothesis.

Here we present a patient with steroid-resistant nephrotic syndrome (SRNS) treated with RTX during a phase of active proteinuria. The clearance of RTX was extremely fast, partly explainable by excessive urinary losses.

## Case report

A 10-year-old Afro-American boy (36 kg), diagnosed with idiopathic nephrotic syndrome for 3 years, was treated initially with prednisolone (1.5 mg/kg/day) and then for a shorter time with mycophenolate mofetil (MMF) partly in combination with cyclosporine. His caretaker refused to continue the advised cyclosporine + MMF treatment or to switch to tacrolimus and only gave him high-dose prednisolone for more than 1 year. In this year he developed steroid resistance. A kidney biopsy—taken shortly before treatment with RTX—showed minimal change nephropathy.

On admission the patient was Cushingoid and had proteinuria with significant peripheral edema, pleural and pericardial effusion, and ascites. Following drainage of the pleural, pericardial, and peritoneal fluid he showed clinical improvement. Laboratory results demonstrated elevated serum creatinine [91 μmol/L; estimated glomerular filtration rate 60 mL/min/1.73 m^2^; normal range 80–120 mL/min/1.73 m^2^ (Schwarz formula)], elevated blood urea nitrogen (8.2 mmol/L; normal range 1.8–6.4 mmol/L), and severe hypoalbuminemia (≤15 g/L; normal range 35–55 g/L). Serum immunoglobulin G (IgG) was extremely low (0.25 g/L; normal range 5.2–15.6 g/L), probably due to non-selective proteinuria. Urinalysis showed severe proteinuria (21.5 g/L; >2220 mg albumin/mmol creatinine), with a low selectivity index of 0.33 [(urine IgG/serum IgG) × (serum albumin/urine albumin)]. The clinical decision to treat the patient with RTX was made in order to achieve quick clinical improvement.

**Fig. 1:**
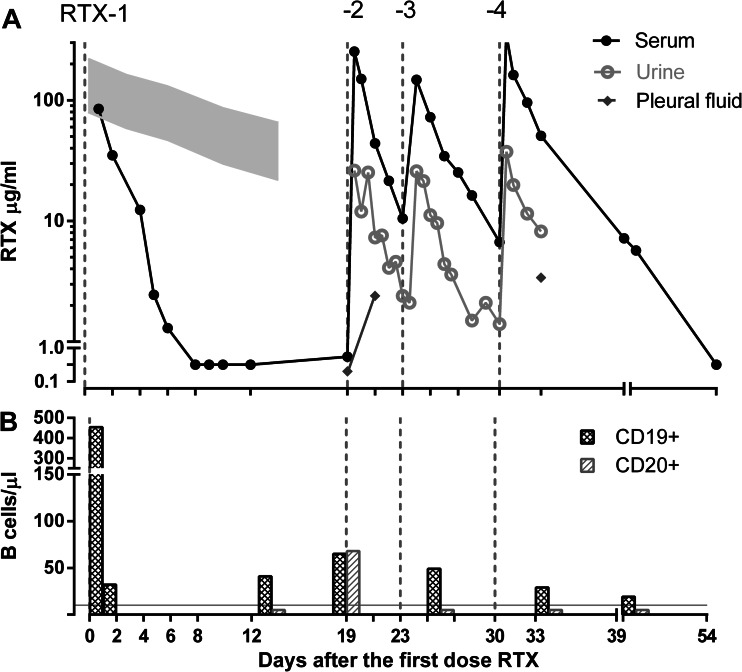
Level of rituximab (RTX) and B cells after initiation of RTX therapy in nephrotic patient. **a** RTX levels in serum (*black line*), urine (*gray line*), and pleural fluid (*diamonds*) over time (days) in relation to four RTX infusions. *Gray area in left upper part* indicates RTX levels of patients receiving RTX post-stem cell transplantation without urinary protein loss. **b** Simultaneous measurements of CD19+ and CD20+ B cells. *Gray line* Our definition of CD20+ B cell depletion of <10 CD20+ B cells/μL

RTX was started at a dose of 375 mg/m^2^, and serum concentrations of RTX and B lymphocyte counts were measured. The concentration of RTX in serum samples was determined using a direct enzyme-linked immunosorbent assay [see Electronic Supplementary Material (ESM)]. RTX concentrations fell very quickly (Fig. 1a), and at 14 days after the initiation of RTX therapy, complete CD20+ B cell depletion was documented (<10 CD20+ B cells/μL) (Fig. 1b); however, as quickly as 1 week later CD20+ B lymphocytes reappeared. The next three RTX doses were given at shorter intervals, with a target serum RTX concentration of >10 μg/L (20, 24 and 31 days after the first dose, respectively). This therapeutic strategy achieved CD20+ B lymphocyte depletion from day 19 to day 39. A few circulating CD19+ B lymphocytes were still present at day 39, but after day 39 B lymphocytes were not tested anymore. The elimination serum half-life of RTX in our patient was <1 day, compared to 20 days in a group of patients receiving RTX post-stem cell transplantation. These latter patients had no urinary protein loss, normal kidney function, and no ascites (C.M. Jol–van der Zijde; unpublished observations). High concentrations of RTX were found in our patient’s urine and pleural fluid (Fig. 1a).

The population pharmacokinetics of RTX was simulated in a two-compartment structure [8, 9], with additional simulations for urinary and pleural fluid loss, using the software package R 2.12 (see ESM Table 2). Urinary clearance, as derived from the measurements of RTX concentrations in the urine and urine production, was at least 25 % of the total clearance, whereas in stem cell transplantation patients without proteinuria, urinary clearance was nil. Pleural clearance, as derived from RTX concentrations in pleural fluid and the drained amount of pleural fluid, was around 2 % of the total clearance (data not shown). However, these losses explained only part of the rapid drop in serum RTX concentration. Antibodies against RTX could not be detected [10].

Because of ongoing proteinuria and ascites, pleural and pericardial effusions after the four doses of RTX, and severe hypertension, an angiotensin-converting enzyme-inhibitor (enalapril), diuretics, and tacrolimus were added to the therapeutic regimen. Four months after the first dose of RTX, prednisolone was tapered and could be stopped. Six months after RTX treatment, the patient still had proteinuria (1000 mg albumin/mmol creatinine), but he was in a stable situation, without edema and with normal blood pressure, under treatment with tacrolimus, ramipril, and hydrochlorothiazide.

## Discussion

To our knowledge, this is the first report of the pharmacokinetic profile of RTX in a patient in a nephrotic state demonstrating excessive urinary loss of RTX. Previous publications have shown that RTX treatment may be less effective for SRNS than for steroid-dependent (SDNS) or frequently-relapsing nephrotic syndrome [2, 4, 5, 11, 12]. In a large retrospective multicenter study of 70 nephrotic patients [4], 41 % (11/27 with SRNS) had minimal change nephrotic syndrome (MCNS), similar to our patient, but no information on the amount of proteinuria was reported by the authors. Of the 27 SRNS patients, 12 (44 %) had a good initial response to RTX, of whom four (33 %) had MCNS and six (50 %) had focal segmental glomeruloscerlosis. Of the 28 patients with SDNS, 23 (82 %) had a good initial response to RTX [4]. In a more recent randomized controlled trial involving 31 patients with SRNS [11], half of the patients were treated with RTX (375 mg/m^2^; two injections, with a 2-week interval between injections). All patients were concurrently treated with prednisolone or cyclosporine. After 3 months of follow-up, responses were similar in both groups. None of the early SRNS patients had a good response. Among six SRNS patients who developed prednisolone resistance months or years after disease onset, those in the RTX group showed some response, especially those who had responded to standard drugs earlier [11]. The poor results in the SRNS group may have been due to the loss of RTX in the urine [2, 5–7].

Few studies have focused on the pharmacokinetics of RTX in patients with nephrotic syndrome [7, 11, 13]. The mean serum half-life of RTX in patients with SDNS is reported to be 14.6 ± 5.2 days (no information was provided by these authors on proteinuria) [13], which is only slightly shorter than the 20 days in patients who received RTX for other indications (i.e., reactivation of Epstein–Barr virus after allogeneic stem cell transplantation; C.M. Jol–van der Zijde, unpublished observations). In contrast, the serum half-life of RTX in our patient was <1 day. Fervenza et al. [7] measured plasma RTX concentrations in 15 patients with severe proteinuria and found that 14 days after RTX infusion the plasma concentration was 17 ± 11 μg/mL. In our patient, the plasma RTX level was already <0.1 μg/mL after 7 days. One possible explanation for these differences in serum half-life and plasma RTX concentrations is that our patient had severe non-selective proteinuria; consequently, RTX, with an approximate molecular weight of 145 kDa, was lost in the urine, causing insufficient RTX exposure to deplete all circulating CD20+ B cells. While the duration of B cell depletion in patients with refractory nephrotic syndrome was previously reported to be approximately 3–5 months [14, 15], in our patient B cells reappeared within 20 days after the first dose of RTX, probably due to the short serum half-life of RTX; additional losses in the pleural effusion and ascites in our patient contributed to the shortening of the serum RTX half-life. Nevertheless, the pharmacokinetics studies in our patient and the simulation studies only partly explain the extremely short serum half-life of RTX. Other factors which could be of importance in this respect are intra-vascular dehydration, stimulation of the metabolism of RTX by the liver due to hypo-albuminemia, and/or an effect of Fcγ gamma receptor polymorphisms [16].

Our initial aim was to treat our patient with one dose of RTX. When B cells reappeared shortly thereafter however, we decided to provide a full treatment with four doses, measuring urinary RTX loss and serum RTX concentrations after each infusion. The timing of each following dose of RTX was chosen to maintain the serum RTX concentration at >10 μg/mL in order to achieve more prolonged B cell depletion. Despite this strategy, stable remission of the nephrotic syndrome was not immediately achieved, and additional treatment modalities were necessary. Although B cell depletion itself is thought to be an important factor affecting the long-term effectiveness of RTX treatment, Guigonis et al. [2] reported that complete B cell depletion is necessary but not sufficient for RTX to be effective in SDNS. The pharmacokinetics of RTX may be affected by the nephrotic state, leading to only a partial depletion of non-circulating B cells (CD20+) despite complete depletion of circulating B cells (CD20+) [2, 5]. On the other hand, Prytula et al. [4] reported that two children without CD19+ B cell depletion showed good clinical response to RTX. Additionally, it has been reported that clinical remission can last longer than the depletion of B cells in the peripheral blood [2], suggesting additional effects of anti-B cell therapy [1, 3].

In conclusion, the serum half-life of RTX can be extremely short in patients with therapy-resistant nephrotic syndrome with non-selective proteinuria, partly because of excessive urinary loss. However, there must be other, as yet unidentified factors that contribute to this extremely short half-life of RTX.

## Electronic supplementary material

ESM 1 (DOC 25 kb)

ESM 2 (GIF 66 kb)

High resolution image (TIFF 4400 kb)
