# Supplementary material for: Pharmacokinetics of rituximab in a pediatric patient with therapy-resistant nephrotic syndrome
Source: Pediatr Nephrol. 2015 Jun 9;30(8):1367–70. doi: 10.1007/s00467-015-3120-8 (PMC4483248; doi:10.1007/s00467-015-3120-8)
Supplement: Supplementary file 1 — (DOC 25 kb) [file 467_2015_3120_MOESM1_ESM.doc]

**Electronic Supplementary Material**

#### RTX measurements

#### RTX in serum was measured with an ELISA. Microtiter plates were coated with 0.5 μg/mL rat anti-RTX (MCA2260; AbD Serotec, Kidlington, United Kingdom). After incubation of standards (range 0.3 – 10 ng/mL) and diluted sera from patients, rabbit anti-human IgG (Fcγ-specific) conjugated to AP (Jackson ImmunoResearch Laboratories) was added; high and low controls were used in each assay. The limit of detection in serum RTX is 0.1 μg/mL.

**Pharmacokinetics**

The population pharmacokinetics of RTX was simulated in a 2-compartment structure, with additional simulations for urinary and pleural fluid loss, using the software package R 2.12 [1]. The presented data contained observations of one single individual, therefore a population approach pharmacokinetic model to estimate the lacking population pharmacokinetic parameters for the urine and pleural compartments could not be developed. In order to describe the data, a sensitivity analysis was performed on this literature-based structural model; fixed literature values and a range of values for remaining pharmacokinetic parameters (e.g. renal and pleural clearance) were used to simulate concentration-time profiles that allowed simultaneous description of observed plasma, urine and pleural rituximab concentrations (see Figure 2).

**Supplementary figure legends**

**Supplementary Figure 1. Simulated RTX concentrations of nephrotic and non-nephrotic patients**

2a: Simulated RTX urine concentrations over time (solid line) and the observations (circles). The dotted lines represent the dosing times.

2b: Simulated RTX plasma concentrations over time in our patient (solid line) and non-nephrotic patients (dashed line) with their observations (circles and triangles, respectively). The dotted lines represent the dosing times.

**Supplementary reference**

1. R Development Core Team (2010) R: A language and environment for statistical computing. R Foundation for Statistical Computing, Vienna, Austria. ISBN 3-900051-07-0, URL <http://www.R-project.org/>
