# Supplementary figures and images for: Pharmacokinetics of rituximab in a pediatric patient with therapy-resistant nephrotic syndrome
Source: Pediatr Nephrol. 2015 Jun 9;30(8):1367–70. doi: 10.1007/s00467-015-3120-8 (PMC4483248; doi:10.1007/s00467-015-3120-8)

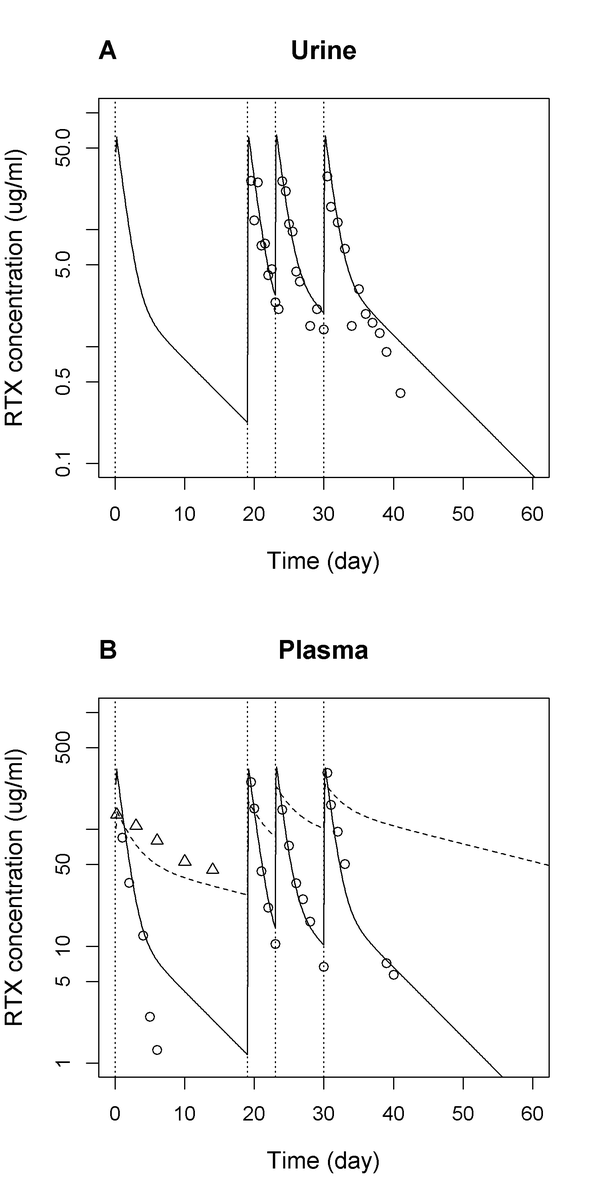

Supplement: Supplementary file 2 — (GIF 66 kb) [file 467_2015_3120_Fig2_ESM.gif]
